# Does Our Profession Confer All the Benefits It Should?

**Published:** 1890-07

**Authors:** E. J. Waye

**Affiliations:** Sandusky, O.


Dr. E. J. Waye, of Sandusky, O., read a paper on

### Does Our Profession as Practiced at the Present Time, Confer upon the Community all the Benefits It Should? If not, Why? And What is the Remedy?

BY E. J. WAYE, D.D.S., SANDUSKY, O.

A somewhat extended experience as a dentist, during which I have had frequent opportunities for conversation with "all sorts and conditions of people," has convinced me that upon no other subjects which in any degree approaches it in importance, a knowledge of which so intimately concerns every individual in the community, does there exist so universal, so deplorable an ignorance as to-day exists concerning the teeth. This ignorance is confined to no grade or condition, it is found everywhere and differs only in degree. From the lowly hut of the laborer, whose daily toil can scarcely provide for those dependent upon him the plainest fare and coarsest clothing, to the palatial dwellings of the rich, the refined, the educated and the fastidious, ignorance concerning the teeth is the rule, and knowledge the exception.

This general condition of ignorance does not refer particularly to the care and attention which should be bestowed upon the teeth; though even here we have by no means reached the highest cultivation. Neither is it the entire community to which attention is directed at this time, but rather to a class which exists in every community. It is not a *general* ignorance concerning the teeth and their care to which we now refer. But rather an ignorance concerning certain phases of their existence, during which the teeth are wholly under the care and supervision of this class, and through whose ignorance, and the neglect resulting therefrom by which their disease, decay, and loss to an extent most deplorable is the inevitable result, and this before the age at which the services of the dentist are deemed necessary.

The victims to this ignorance are the children. The injury inflicted occurs during childhood. And the almost wholly innocent and ignorant cause of what, to the children, is a life-long deprivation, an irreparable injury, is the mothers. This is certainly a most grave and solemn indictment. And what to me, seems the most sad and lamentable feature in the case is that, unless my observation for years is most egregiously at fault, the charge is altogether demonstratable.

In holding this opinion do not for one moment believe me capable of believing that a mother either knowingly or willingly would permit what she believed to be an injury to her child. It is this very unconsciousness of danger in which lurks the peril, and it is ignorance which accounts for the lack of consciousness.

In the life of every child occurs two most important epochs in each of which certain mysterious processes are carried forward to completion. The first of these occurs in infancy. Its first visible appearance being about the fifth month, and it continues usually through the second year, at the end of which the little mouth is filled with the temporary set of twenty teeth. This is known as first dentition. This period of two years is one which, to the mother, is fraught with much anxiety and apprehension. It is not unfrequently attended with violent symptoms, which sometimes result fatally, and when safely passed, and the little semi-circle of pearls in position, the joy of the mother knows no bounds. She may now rest in peace, her nightly vigils ended, and the grim spectre which for long weary months has haunted her dreams is now at rest, and peace and happiness at length resume their supremacy.

Then comes the period of second dentition. Of this the mother knows but little beyond the fact that there are two sets of teeth. That the first of temporary teeth will be shed, and a second and larger set will take their places. As this process does not begin in a long time, is attended with no danger and but little inconvenience, all anxiety concerning it may deferred till such time as there is a reason for it.

As I am now talking to dentists no account of what occurs between this period and the first visit to the dentist will be given, but instead, an imaginary visit of the mother to the dentist will, I think, better serve to illustrate the ignorance of the one and its results upon the others. Under ordinary circumstances no particular trouble may have been experienced with the teeth up to the tenth or twelfth year. At this age many of the temporary teeth have disappeared and been replaced by those of the permanent set. The sixth year permanent molars are in position and quite probably those of the twelfth year also.

The mother whom we will suppose to belong to that respectable class educated in our common schools, one who reads the papers and books, and possess a full average amount of intelligence and general knowledge of every day affairs, enters the office of the dentist with her two children, one of them nine, and the other thirteen years of age. "Doctor, my little girl has been having the toothache." "Ah!" "Yes, in two of her first teeth." (Emphasis on "first.") You seat her in the chair and proceed to make an examination, which shows the aching teeth to be two of the six year molars. One in the upper, and on the opposite side, one in the lower jaw. You find decay, a good deal of it, crown approximal cavities in both, the teeth much broken down, the pulps exposed, and such a condition of things in the mouth as clearly indicates an utter disregard of cleanliness or attention of any kind. Food in all stages of decomposition is around and between the teeth, and under the free edges of the gums.

"Those are the first teeth are they not, Doctor?" "Yes, madam, and last as well unless supplied by the dentist." "Won't she get new ones if these are extracted?" "No, madam, these teeth erupt but once, they are the first permanent grinding teeth, or molars, as dentists term them." "Why, these are the first teeth, I am sure they have never been shed." Very true, and as I said they are also the last teeth the child will ever have in that place." "Well, now I thought all the teeth were shed. What can you do for them?" Each one can answer that question as his own ideas of similar cases may prompt, but I incline to the opinion that most of us will think that those two most important and valuable teeth will be lost ere long.

Her boy, aged 13, has also been a sufferer from toothache, and in his case it is not only two of the sixth year molars, but those of the twelfth year also, which have recently made their appearance, the pulp of one of them being exposed, and another decaying. Two of the sixth year molars are too much broken down to admit of being filled successfully. The mother looks on during the examination, listens to the diagnosis, and such remarks concerning the injury which the loss of these important organs is certain to entail upon her children, (and should her dentist be an earnest, conscientious man, and not too much hurried with work), possibly also a brief sketch of the process of dentition, with a few kindly words as to the duty of the mother in the case.

And then in a voice in which sorrow seems blended with accusation, she exclaims: "But, Doctor, I knew nothing of all these things ; my mother taught me nothing whatever concerning my teeth. Probably knew nothing herself. Do you wonder that my children's teeth were neglected? I was ignorant and who was there to teach me?"

Perhaps no dentist present has heard from the lips of any dentist, either a confession like the above, or the interrogatory which followed. But that all have had presented for their inspection mouths in which existed conditions similar to the ones described, perhaps none will deny; mouths in which the teeth themselves afforded evidence irrefragable of total neglect. And if neither the confession nor the question has been elicited, it was not because of any superior knowledge which might render such teaching necessary, but rather, of an indifference born of ignorance, which prevented her from bestowing upon the subject any proper consideration, or of a neglect the most culpable and inexcusable.

Through the period of infancy and childhood almost the entire care and management of the teeth is intrusted to the mother, and whatever attention they may receive is the result of her supervision. Now, if the mother has so little knowledge of second dentition as to mistake the molars of the permanent set for the temporary teeth, which so far as my observation and experience goes is almost universally the case, and thus these priceless teeth through neglect become so diseased and decayed as when first seen by the dentist to be past preservation, upon whom rests the responsibility?

If the dentist, who beyond every other individual possesses that knowledge, a part of which should be imparted to the mother, the results of whose ignorance he witnesses daily, shall stand listlessly by, making no effort for its alleviation, upon whom shall she depend for that light and knowledge, the lack of which is now working injury to her child?

With you is the answer. I, of course, have my own ideas, but having already too long trespassed upon your kind indulgence, I will leave for others its future consideration.

---

Owing to the lateness of the hour these papers were passed without discussion. On motion, half past one o'clock Thursday was set apart for a visit to the State Prison.

Dr. Crouse said he wanted to thank the association for the hearty manner in which it had received him. He had received twenty-five members since he came, and he thought Michigan would redeem herself. He wished the association would appoint three good, live men to assist him in the work of the Dental Protective Association in this State.

Dr. Taft asked that the papers that had been read before the association be published in the DENTAL REGISTER and *Ohio Journal of Dental Science*, together with a report of the discussions that were being made for the journals.

Adjourned until 9 A. M. to-morrow.

[TO BE CONTINUED.]

